# The Surface Layer Homology Domain-Containing Proteins of Alkaliphilic *Bacillus pseudofirmus* OF4 Play an Important Role in Alkaline Adaptation via Peptidoglycan Synthesis

**DOI:** 10.3389/fmicb.2018.00810

**Published:** 2018-05-01

**Authors:** Shun Fujinami, Masahiro Ito

**Affiliations:** ^1^Bio-Nano Electronics Research Centre, Toyo University, Kawagoe, Japan; ^2^Department of Chemistry, College of Humanities and Sciences, Nihon University, Tokyo, Japan; ^3^Graduate School of Life Sciences, Toyo University, Tokyo, Japan

**Keywords:** alkaliphiles, alkaline environmental adaptation mechanisms, peptidoglycan synthesis, S-layer protein, SLH domain, *Bacillus*

## Abstract

It is well known that the Na^+^ cycle and the cell wall are essential for alkaline adaptation of Na^+^-dependent alkaliphilic *Bacillus* species. In *Bacillus pseudofirmus* OF4, surface layer protein A (SlpA), the most abundant protein in the surface layer (S-layer) of the cell wall, is involved in alkaline adaptation, especially under low Na^+^ concentrations. The presence of a large number of genes that encode S-layer homology (SLH) domain-containing proteins has been suggested from the genome sequence of *B. pseudofirmus* OF4. However, other than SlpA, the functions of SLH domain-containing proteins are not well known. Therefore, a deletion mutant of the *csaB* gene, required for the retention of SLH domain-containing proteins on the cell wall, was constructed to investigate its physiological properties. The *csaB* mutant strain of *B. pseudofirmus* OF4 had a chained morphology and alkaline sensitivity even under a 230 mM Na^+^ concentration at which there is no growth difference between the parental strain and the *slpA* mutant strain. Ultra-thin section transmission electron microscopy showed that a *csaB* mutant strain lacked an S-layer part, and its peptidoglycan (PG) layer was disturbed. The *slpA* mutant strain also lacked an S-layer part, although its PG layer was not disturbed. These results suggested that the surface layer homology domain-containing proteins of *B. pseudofirmus* OF4 play an important role in alkaline adaptation via peptidoglycan synthesis.

## Introduction

The typical Na^+^-dependent alkaliphilic *Bacillus* species require Na^+^ for growth and motility (Krulwich et al., 2001, 2011; Ito et al., 2011). In *Bacillus pseudofirmus* OF4, the growth at pH 7.5 requires a higher Na^+^ concentration (at least 25 mM and, optimally more than 50 mM) than growth at pH 10.5 (10 mM Na^+^), and the motility at pH 7.0 also requires a higher Na^+^ concentration (100 mM) than motility at pH 10 (5 mM Na^+^) (Gilmour et al., 2000; Fujinami et al., 2007b). The well-characterized alkaline adaptation system, which is also called the Na^+^ cycle, is composed of Na^+^ efflux (e.g., Na^+^/H^+^ antiporters) and influx (e.g., voltage-gated Na^+^ channels, Na^+^-dependent flagellar motor stators, and Na^+^-dependent solute transporters) components (Ito et al., 2004a,b; Fujinami et al., 2007a,b; Morino et al., 2014).

Since protoplasts of Na^+^-dependent alkaliphilic *Bacillus halodurans* C-125 are unstable in an alkaline environment, the cell wall is also considered to be important for alkaline adaptation (Aono et al., 1992). The cell wall of *B. halodurans* C-125 consist of peptidoglycan (PG) and non-peptidoglycan components. The PG is the A1γ type, identical to that of neutrophilic *B. subtilis* (Aono et al., 1984). The cell wall has two major kinds of acidic polymers called teichuronic acid (TUA) and teichuronopeptide (TUP) (Aono, 1985). TUP is a polymer in which an acidic polypeptide binds covalently to polyglucuronic acid. The abundance of the acidic surface polymers, as well as the accompanying high anionic charge and proton accumulation around the cell, are thought to prevent hydroxide ion penetration (Aono et al., 1992). Therefore, TUA and TUP are considered to contribute to cell adaptation to an alkaline environment. It was reported that the amounts of TUA and TUP are enhanced in cells grown at an alkaline pH, as compared to those grown at neutral pH (Aono, 1985), and the mutants deficient in TUA and TUP have lost alkaliphilicity (Aono and Ohtani, 1990; Aono et al., 1995, 1999; Ito and Aono, 2002).

**Figure 1:**
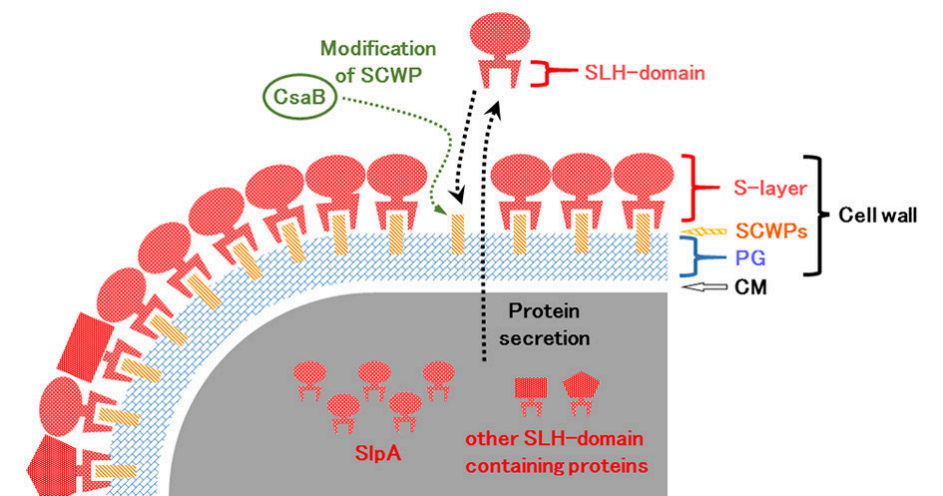
Schematic diagram of the putative surface layer structure of *B. pseudofirmus* OF4. The cell wall of *B. pseudofirmus* OF4 is composed of PG, SCWPs, and an S-layer. It was suggested that the SLH domain-containing proteins are translated in the cytoplasm and secreted to the extracellular space by a protein secretion system. Secreted SLH domain-containing proteins are anchored to SCWPs that are modified by the polysaccharide pyruvyl transferase CsaB via the SLH domain. S-layer, surface layer; SLH, surface layer homology; SCWPs, secondary cell wall polymers; PG, peptidoglycan; CM, cytoplasmic membrane.

In *B. pseudofirmus* OF4, the lag phase of a mutant deficient in the surface layer (S-layer) protein A (SlpA) was increased at pH 11, especially under low Na^+^ concentrations (i.e., 5 mM), and lacked an S-layer region (Gilmour et al., 2000). A schematic diagram of the putative S-layer structure of *B. pseudofirmus* OF4 is shown in Figure 1. The genome sequence of *B. pseudofirmus* OF4 codes for 17 S-layer homology (SLH) domain-containing proteins, including SlpA (Table 1). Although these genes do not have an operon structure, their gene products are thought to be retained in the cell surface in the same manner via its SLH domain. Most of the gene products were presumed to have three SLH domains immediately after the N-terminal signal peptide or at the C-terminus. It was proposed that the SLH domain binds to secondary cell wall polymers (SCWPs) (Schäffer and Messner, 2005). The SlpA, which has three SLH domains, is the most abundant protein in the cell wall of *B. pseudofirmus* OF4. The isoelectric points (p*I*) of the extracellular and cell wall proteins of alkaliphiles are reported to be relatively low (Janto et al., 2011). The SlpA protein, which has a p*I* of 4.36 (without signal peptide), contains few arginine and lysine residues. It is thought that the SlpA protein also causes a proton accumulation and prevention of hydroxide ion penetration, similarly to TUA and TUP (Gilmour et al., 2000; Krulwich et al., 2011; Krulwich and Ito, 2013).

**Table 1:** SLH domain-containing proteins of *B. pseudofirmus* OF4 suggested from the genome sequence.

**Gene name**		**UniprotKB accession number**	**Protein function**	**Mass**	**Graphicval view of SLH domain**
BpOF4_05935		D3FZK7	SLH domain-containing esterase	74,603	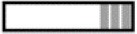
BpOF4_16655	*ushA*	D3FQK4	Protein ushA (Two S-layer domains, UDP-sugar hydrolase, 5′-nucleotidase)	70,385	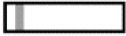
BpOF4_06105	*lytC*	D3FZP1^*^	N-acetylmuramoyl-L-alanine amidase containing SLH domains	50,569	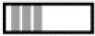
BpOF4_05925		D3FZK5	5′-nucleotidase and SLH domain-containing protein	89,763	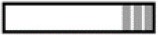
BpOF4_06055	*amyA*	D3FZN1	Alpha-amylase/pullulanase (1,4-alpha-D-glucan glucanohydrolase, Pullulanase) with SLH domain	293,461	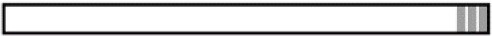
BpOF4_06130	*amyA2*	D3FZP6	Alpha-amylase/pullulanase (1,4-alpha-D-glucan glucanohydrolase, Pullulanase)	83,651	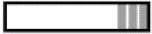
BpOF4_20104		D3G0Z4	SLH domain-containing hydrolase, putative beta-lactamase	59,025	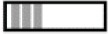
BpOF4_05815		D3FZI3^*^	SLH domain protein, peptidoglycan NAcGlcam'ase	71,137	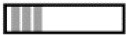
BpOF4_06075	slpA	D3FZN5	S-layer glycoprotein, contains three N-terminal SLH domains	96,883	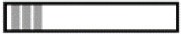
BpOF4_06080		D3FZN6^*^	Cell wall-associated hydrolase containing three SLH domains	36,064	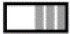
BpOF4_05835		D3FZI7	SLH domain, hydrolase	39,553	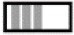
BpOF4_06085		D3FZN7	Alkaline serine proteinase and SLH-domain protein	67,576	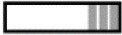
BpOF4_05820		D3FZI4	SLH domain protein	82,212	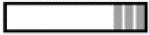
BpOF4_05825		D3FZI5	SLH-domain protein	24,467	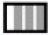
BpOF4_05940		D3FZK8	Uncharacterized protein	40,418	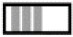
BpOF4_21149		D3G1J9	S-layer like protein	45,236	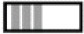
BpOF4_05920		D3FZK4	Putative S-layer protein	44,960	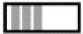

*This table was prepared from data extracted from UniprotKB*.

* *Putative SLH domain-containing peptidoglycan hydrolases The white box represents the putative gene product. The N-terminus is on the left side and the C-terminus is on the right side. Gray box shows the location of SLH domain in putative gene product*.

The functions of the SLH domain-containing proteins other than SlpA are not well known. We hypothesized that SLH domain-containing proteins, other than SlpA, involved in alkaline adaptation. However, investigating the effect of numerous SLH domain-containing proteins on alkaline adaptation one by one requires an enormous amount of time, and there is also the possibility that it will not appear as a phenotype unless multiple genes are deleted. Therefore, a *csaB* deletion mutant of *B. pseudofirmus* OF4 was constructed. CsaB itself does not contain the SLH domain, but it was reported to act as an SCWP modification polysaccharide pyruvyl transferase (Kern et al., 2010), and it is required for the retention of SLH domain-containing proteins to the SCWPs of the *Bacillus anthracis* cell wall (Mesnage et al., 2000). It is thought that the SLH domain binds to the CsaB-catalyzed pyruvylation moieties of SCWP. Therefore, even in *B. pseudofirmus* OF4, the CsaB mutant strain was considered to show the phenotype in the case where the cell wall had no SLH domain-containing proteins at all.

In this study, we report microscopic observations of the cell morphology, cell wall components, and cell growth at different pH values of *B. pseudofirmus* OF4 and its S-layer protein mutant strains to elucidate the effects of SLH domain-containing proteins on alkaline adaptation and cell morphology.

## Materials and methods

### Bacterial strains and media

The bacterial strains and plasmids used in this study are listed in Table 2. *Escherichia coli* strains were grown routinely in lysogeny broth (LB) medium (Sambrook et al., 1989). *B. pseudofirmus* OF4-811M and its derivative cells were grown in alkaline complex medium (pH 8.0, 9.0, 10, and 11) at 30°C with shaking at 200 rpm (Fujinami et al., 2007b). The Na^+^ concentration of alkaline complex medium is 230 mM in all cases.

**Table 2:** Bacterial strains and plasmids used in this study.

**Strain and plasmid**	**Genotype and description**	**Source and reference**
***E. coli*** **STRAINS**		
DH5αMCR	F- *mcrAΔ1 (mrr-hsd RMS-mcrBC) Φ80dlacZ Δ(lacZYAargF) U169 deoR recA1 endA1 supE44 λthi-1 gyr-496 relA1*	Stratagene
XL1-Blue MRF'	*Δ(mcrA)183 Δ(mcrCB-hsdSMR-mrr)173 endA1 supE44 thi-1 recA1 gyrA96 relA1 lac [F'proAB lacIqZDM15 Tn10 (Tetr)]*	GIBCO/BRL
***BACILLUS PSEUDOFIRMUS*** **OF4 STRAINS**		
811M	wild-type, Met^−^	Clejan et al., 1989
RG21	811MΔ*slpA*::Spc^R^	Gilmour et al., 2000
CS54	811M, *csaB* deletion	This study
CS54-R	CS54, *csaB* restored at the native location	This study
**PLASMIDS**		
pGEM7Zf(+)	Cloning vector Amp^R^	Promega
pGEMC1	pGEM7Zf(+)+Δ*csaB*	This study
pG^+^host4	Temperature-sensitive vector Erm^R^	Appligene
pG4C4	pG^+^host4+Δ*csaB*	This study
pMW118	Cloning vector Amp^R^	Nippon gene
pMWCR	pMW118+*csaB*	This study
pG4CR	pG^+^host4+*csaB*	This study

### Construction of the *csaB* deletion strain CS54 and its restoration strain CS54-R

A DNA fragment containing the upstream and downstream regions of the *csaB* gene was obtained by the gene SOEing (gene splicing by overlap extension) method (Horton, 1997) using the primers listed in Table 3. Two independent polymerase chain reactions (PCRs) were performed with *B. pseudofirmus* OF4-811M chromosomal DNA as the template and the primer pairs CS1F/CS2R-SS and CS3F-SS/CS4R. The two purified PCR products were used as templates for a second PCR with the primer pair CS1F and CS4R. The purified product of this reaction was cloned into the *Sma*I digested plasmid pGEM7Zf(+), which yielded pGEMC1. The mutation-free pEMC1 insert was digested with the endonucleases *Kpn*I and *Bam*HI, and the purified inserted DNA fragment was cloned into the *Kpn*I- and *Bam*HI-digested plasmid pG^+^host4, yielding pG4C4, which was then transformed into *B. pseudofirmus* OF4-811M protoplasts. The protocol of the protoplast transformation and isolation of single and double crossover candidates was previously reported (Ito et al., 1997; Fujinami et al., 2007a). Among the erythromycin-sensitive double crossover candidates, a *csaB* deletion was confirmed by PCR with the primer pairs CS0F/CS4R and CS1F/CS5R. The *csaB* deletion strain was designated CS54.

**Table 3:** Primers used in this study.

**Primer**	**Sequence (5′-3′)**	**Accession number and corresponding sequence (nt)**
CS0F	GAAGATAGAAATACCGGGGC	CP001878.2 [3417065 → 3417046 (minus strand)]
CS1F	ATAGACCTAGCATTAAAGGG	CP001878.2 [3416882 → 3416863 (minus strand)]
CS2R-SS	TCTTGGTCCTTTATATACCCT CCCGGGTCATCTATTCATCC ACCTCTT	CP001878.2 [3415087 → 3415107) CP001878.2 (3416163 → 3416183]
CS3F-SS	AAGAGGTGGATGAATAGATGA CCCGGGAGGGTATATAAAGG ACCAAGA	CP001878.2 [3416183 → 3416163 (minus strand)] CP001878.2 [3415107 → 3415087 (minus strand)]
CS4R	CCATCAATTAAGTTAATGGC	CP001878.2 (3414387 → 3414403)
CS5R	TATCCTAAGAATAAGGCCCC	CP001878.2 (3414195 → 3414214)

*Extra nucleotides that were added to introduce restriction sites for other experiments are underlined*.

For restoration of the *csaB* gene at its native location, a DNA fragment containing the *csaB* gene was obtained by PCR with *B. pseudofirmus* OF4-811M chromosomal DNA as the template and the primer pair CS1F and CS4R. The purified product of this reaction was cloned into *Sma*I-digested pMW118, which yielded pMWCR. The mutation-free pMWCR insert was digested with endonucleases *Kpn*I and *Bam*HI and the purified *csaB* fragment was cloned into a *Kpn*I- and *Bam*HI-digested pG^+^host4, yielding pG4CR, which was transformed into *B. pseudofirmus* OF4-CS54 protoplasts. The protocol for the isolation of single and double crossover candidates was previously reported (Ito et al., 1997). From the restoration strain candidates, which were grown in alkaline complex medium (pH10), *csaB* restoration at its native location was confirmed by PCR using the primer pairs CS0F/CS4R and CS1F/CS5R. The *csaB* restoration strain was designated CS54-R.

### Microscopic observation of cell wall synthesis of *B. pseudofirmus* OF4 by fluorescent vancomycin staining

*Bacillus pseudofirmus* OF4-811M and its derivative cells were grown at pH 8.0, as described above, and then stained with afluorescent vancomycin staining method (Tiyanont et al., 2006) modified for alkaliphilic *Bacillus* species cells, as described by Fujinami et al. (2011). Fluorescence microscopic images were obtained with a Leica FW4000 Fluorescence Workstation (Leica Microsystems AG, Heerbrugg, Switzerland) and processed with Photoshop CS software (Adobe Systems Incorporated, San Jose, CA, USA).

### Sodium dodecyl sulfate polyacrylamide gel electrophoresis (SDS-Page) of proteins of *B. pseudofirmus* OF4

*Bacillus pseudofirmus* OF4-811M and its derivative cells were grown at pH 8.0, as described above. Then, 50 mL of culture was centrifuged at 7,000 g for 5 min at 4°C and separated into supernatant and cell fractions.

The remaining cells were removed from the supernatant fraction by centrifugation at 7,000 g for 5 min at 4°C. Then, the secreted proteins were precipitated with 10% trichloroacetic acid for 30 min on ice and then centrifuged at 16,000 g for 15 min. The precipitates were washed twice with ethanol and then suspended in 200 μL of homogenization buffer (50 mM NaCl, 2.5 mM MgCl_2_, 25 mM K_2_HPO_4_, corrected to pH 7.0 with HCl).

From the cell fraction, protoplasts were prepared, as described by Aono et al. (1992). The cells were washed twice with 3 mL of a selective medium for the isolation of *Megasphaera* and *Pectinatus*, designated SMMP (Chang and Cohen, 1979). Then, 0.01 vol. of 1% (w/v) lysozyme solution was added to the suspension. Protoplast formation at 37°C was monitored microscopically. The protoplasts were centrifuged at 700 g for 10 min at 10°C and separated into cell wall and protoplast fractions. The remaining protoplasts were removed from the cell wall fraction by centrifugation at 700 g for 10 min at 4°C. Then, the cell wall proteins were precipitated with 10% trichloroacetic acid for 30 min on ice and centrifuged at 16,000 g for 15 min. The precipitates were washed twice with ethanol and then suspended in 200 μL of homogenization buffer.

Each protein solution was mixed with an equal volume of sample buffer. A 20-μL aliquot of each sample was separated by 7.5% Tricinre-SDS-PAGE (Schägger and von Jagow, 1987) and analyzed by Coomassie Brilliant Blue staining.

### Ultrathin section transmission electron microscopy (TEM)

*Bacillus pseudofirmus* OF4-811M and its derivative cells were grown at pH 8.0, as described above. The thin section TEM methods described by others (Sleytr et al., 1988; Gilmour et al., 2000) were adapted for this study. Cells were pre-fixed in 1% osmium tetroxide in 0.1 M cacodylate buffer (CB) (pH 7.2) at 4°C for 2 h. The pre-fixed samples were washed three times with 0.1 M CB (pH 7.2) and then fixed with 2.5% glutaraldehyde-4% tannic acid in 0.1 M CB (pH 7.2) at 4°C for 2 h. The fixed samples were washed three times with 0.1 M CB (pH 7.2) and then embedded in 2% Noble agar. After solidification, the agar block was cut into small cubes (<1 mm^3^) and post-fixed with 1% osmium tetroxide in 0.1 M CB (pH 7.2) at 4°C for 18 h. The post-fixed samples were washed twice with 0.1 M CB (pH 7.2) and then dehydrated stepwise according to the following procedures: 50% ethanol at 4°C for 5 min (twice), 70% ethanol at 4°C for 5 min (twice), 90% ethanol at room temperature for 5 min (twice), 95% ethanol at room temperature for 5 min (twice), 100% ethanol at room temperature for 10 min (twice), and propylene oxide at room temperature for 10 min (twice). The dehydrated samples were infiltrated with a 1:1 solution of propylene oxide: embedding medium for 3.5 h and then epoxy resin for 18 h at room temperature. The embedding medium consisted of 12.5 mL of Epon 812, 7.5 mL of Araldite M, 27.5 mL of dodecenylsuccinic anhydride, 1.5 mL of dibutyl phthalate, 0.75 mL of 2,4,6-tri(dimethylaminomethyl)phenol (Nisshin EM Co., Ltd., Tokyo, Japan). To embed the samples, each was transferred to an embedding capsule filled with the embedding medium and incubated at 40°C for 4 days and then at 60°C for 2 days. The blocks were trimmed and sectioned with an ultramicrotome with diamond knives. The ultrathin sections were post-stained with TI blue (Nissin EM Co., Ltd.) at room temperature for 10 min and then washed four times with water. The ultrathin sections were further post-stained with 0.4% lead citrate staining solution at room temperature for 10 min and then washed four times with water. The prepared ultrathin sections were observed using a JEM-2100 electron microscope (JEOL Ltd., Tokyo, Japan) at an acceleration voltage of 100 kV.

The thicknesses of the S-layer, PG, and cell wall (S-layer plus PG) in the microphotograph were measured. The thickness of each cell wall component was measured 10 times at 10 nm intervals using three cells.

### Relative quantification of the amount of DNA of *B. pseudofirmus* OF4-811M and its derivatives cultured at several pH values

*Bacillus pseudofirmus* OF4-811M and its derivative cells were grown on alkaline complex medium (pH 8.0) overnight at 30°C with shaking at 200 rpm. A 1-mL aliquot of culture was then inoculated into 50 mL of fresh alkaline complex medium at pH 8.0, 9.0, 10, or 11 and grown at 30°C with shaking at 200 rpm for 16 h. The whole culture was thoroughly stirred with a vortex mixer, of which 2 mL was used for relative quantification of DNA, according to the method described by Ceriotti (1952). The values were ascertained as a ratio relative to that of *B. pseudofirmus* OF4-811M grown at pH 10, set as 1.0. All results are reported as the averages of three independent experiments.

## Results

### The *B. pseudofirmus* OF4 *csaB* mutant shows chained morphology and alkali-sensitivity

To investigate the physiological functions of SLH domain-containing proteins of *B. pseudofirmus* OF4, a deletion mutant of the *csaB* gene (CS54 strain) and its restoration strain (CS54-R strain) were constructed, as described above. The parental 811M strain (wild type), *slpA* mutant RG21 strain (Gilmour et al., 2000), and CS54-R strain formed typical colonies on agar plates of alkaline complex medium (pH 8 and 10). On the other hand, the CS54 strain did not form colonies on agar plates of alkaline complex medium at pH 10 after overnight incubation, but rather lower viscosity colonies on agar plates of alkaline complex medium at pH 8. In liquid medium, the CS54 strain tended to grow as a macroscopically visible cluster of cells at pH 8.0 even with shaking at 200 rpm, and did not grow at pH 10 (Supplementary Figure S1). Therefore, the 811M strain and its derivative mutants were grown at pH 8.0, as described above. To analyze the details of the cell clusters, microscopic analyses were conducted, which revealed that the morphology of the 811M, RG21, and CS54-R strains were typically rod-shaped, while that of the CS54 strain was chained rod-shaped (Supplementary Figure S2). Nucleoids were observed in the chained rod-shaped cells of the CS54 strain (Supplementary Figure S3).

**Figure 2:**
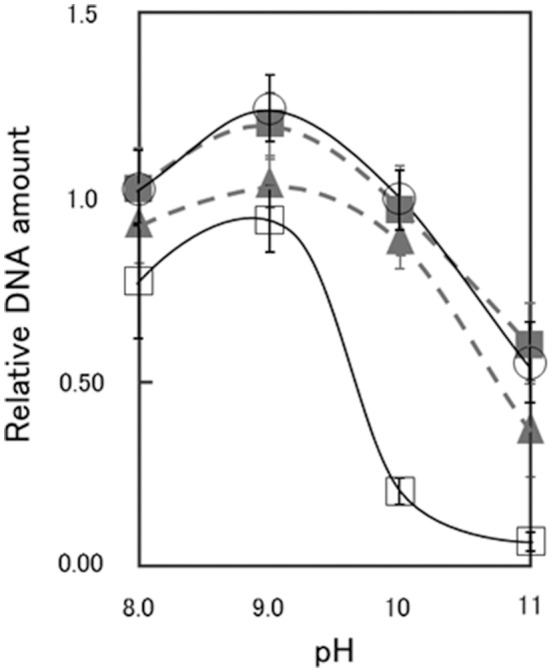
Relative DNA amount of *B. pseudofirmus* OF4-811M and its derivatives grown at several pH values. *B. pseudofirmus* OF4-811M and its derivatives were grown for 16 h at several initial pH values and the DNA was quantified according to the method described by Ceriotti (1952). The black solid line and open circles show data for the 811M strain, the gray dashed line and filled triangles show data for the RG21 strain, the black solid line and open squares show data for the CS54 strain, and the gray dashed line and filled squares show data for the CS54-R strain. The values were ascertained as a ratio relative to that of *B. pseudofirmus* OF4-811M grown at pH 10, set as 1.0. All results are reported as the averages of three independent experiments. The error bars indicate the standard deviations of the values.

As shown in Supplementary Figures S1, S2, the CS54 strain grows in a chained rod-shaped in a liquid medium, and the colonies formed by spreading it on an agar medium are not considered to be derived from one cell. Therefore, it was thought that growth could not be compared by viable cell count. As shown in Supplementary Figure S3, it was suggested that chromosome partitioning occurred in each cells of the CS54 strain, and there was no anucleated cell. Therefore, it was thought that growth could be compared by the relative quantification of the amount of DNA. As shown in Figure 2, there was no significant difference in the amount of DNA among the 811M, RG21, and CS54-R strains at each pH value. In the CS54 strain, a significant decrease of the amount of DNA was observed at pH 9, 10, and 11.

### A deletion of the S-layer and disturbance of PG synthesis were observed in the *B. pseudofirmus* OF4 *csaB* mutant

**Figure 3:**
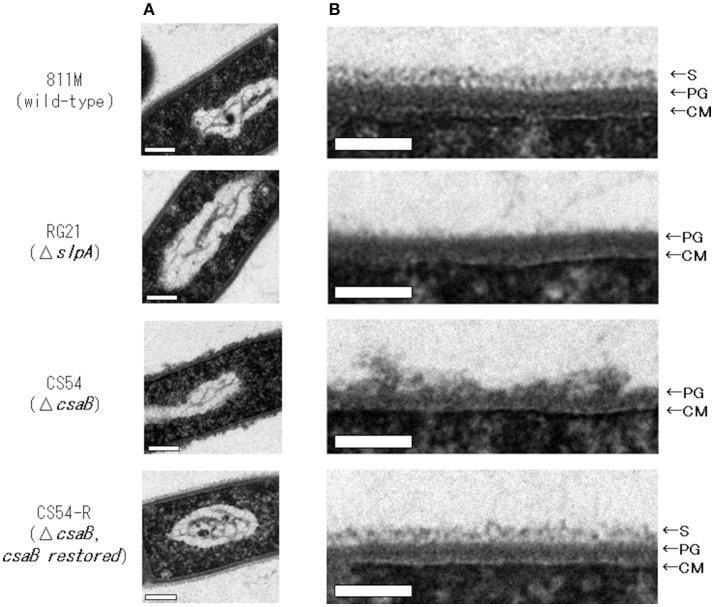
Ultrathin section TEM of *B. pseudofirmus* OF4-811M and its derivative cells. *B. pseudofirmus* OF4-811M and its derivatives were grown at pH 8.0. Ultrathin sections were obtained, as described in the Materials and Methods section, and observed by TEM. Whole cell image **(A)** and an enlarged image of the cell surface region **(B)**. Representative images of the 811M, RG21, CS54, and CS54-R strains are shown. S-layer, surface layer; PG, peptidoglycan; CM, cytoplasmic membrane. Scale bar: 100 nm.

To observe the cell wall of the 811M strain and its derivative cells, thin section TEM was carried out (Figure 3) and the thicknesses of the S-layer, PG, and cell wall (S-layer plus PG) were measured from the photographs (Table 4). The 811M and CS54-R strains have an S-layer as the outermost cell envelope component. The CS54 strain lacked an S-layer and its PG was disturbed, while the RG21 strain also lacked an S-layer but its PG was not affected.

**Table 4:** The thickness of each cell wall component in each of the strains.

	**S-layer (nm)**	**Peptidoglycan (nm) (PG)**	**Cell wall (nm) (S-layer + PG)**
811M	25 ± 3.4	31 ± 4.3	56 ± 5.2
RG21	N.D.	33 ± 3.2	33 ± 3.2
CS54	N.D.	53 ± 19	53 ± 19
CS54-R	25 ± 3.7	30 ± 4.0	55 ± 5.9

*N.D., not detected*.

*The thickness of each cell wall component was measured 10 times at 10 nm intervals using three cells*.

**Figure 4:**
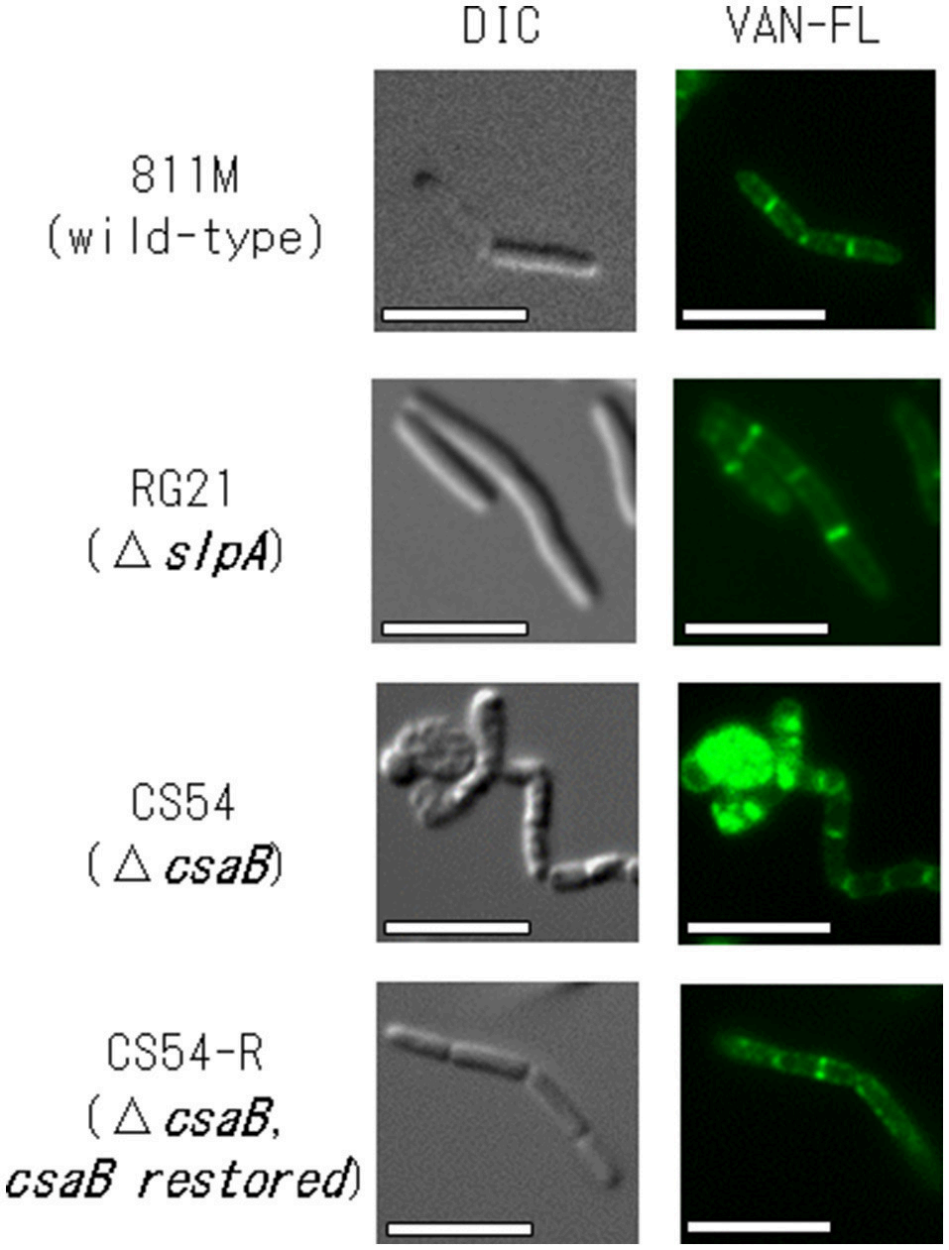
Microscopic observation of the new peptidoglycan of *B. pseudofirmus* OF4-811M and its derivatives *B. pseudofirmus* OF4-811M and its derivatives were grown at pH 8.0. The insertion of new peptidoglycan was stained with FM4-64 and observed by fluorescence microscopy. Representative images of the 811M, RG21, CS54, and CS54-R strain are shown. DIC, differential-interference contrast microscopy; VAN-FL vancomycin, fluorescent vancomycin staining. Scale bar: 5 μm.

To confirm the insertion of new PG in the 811M strain and its derivative cells, the staining patterns of the cells were compared using fluorescent derivatives of the peptidoglycan-binding antibiotic vancomycin (Figure 4). If PG synthesis of strain CS54 was disturbed, the site of insertion of new PG was considered to be different from that of the 811M strain. Bright signals were observed at mid-cell and the poles of the cells in every strain. However, an additional bright signals, indicates the site where insertion of PG above the normal level occurs, as observed in the clustered cells of the CS54 strain.

### SDS-Page analysis revealed that the cell wall of the CS54 strain does not contain a protein of about 94 kDa

**Figure 5:**
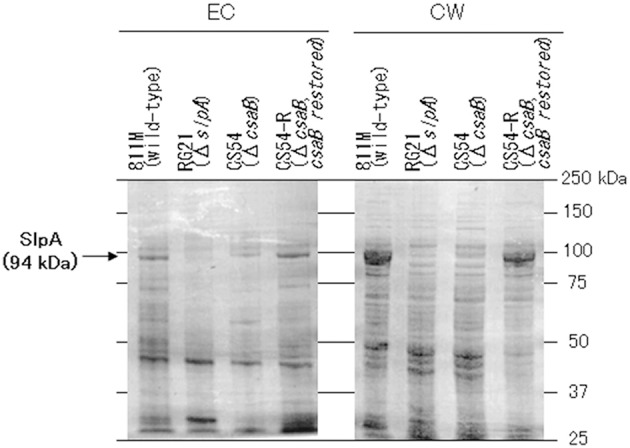
SDS-PAGE of the extracellular and cell wall protein fractions of *B. pseudofirmus* OF4-811M and its derivatives. SDS-PAGE of the EC and CW fractions of *B. pseudofirmus* OF4-811M and its derivatives was performed. Proteins at about 94 kDa, corresponding to the size of SlpA, is indicated with arrows. EC, extracellular protein; CW, cell wall protein.

To confirm expression of the SLH domain-containing proteins, the extracellular and cell wall protein fractions were obtained from the 811M strain and its derivatives, and analyzed by SDS-PAGE (Figure 5). In the 811M and CS54-R strains, strong bands were detected at about 94 kDa, in the cell wall protein fraction, corresponding to the size of the SlpA protein. These bands were also detected in the extracellular protein fraction. In the RG21 strain, no bands of about 94 kDa were detected in both fractions. In the CS54 strain, no bands of about 94 kDa were detected in cell wall fraction, and a weak band was detected at about 94 kDa in the extracellular protein fraction. In all cases, no bands of about 40 kDa, corresponding to the size of CsaB protein, were detected.

## Discussion

In this study, we constructed a deletion mutant of the *csaB* gene, required for the retention of SLH domain-containing proteins on the cell wall (Mesnage et al., 2000), of *B. pseudofirmus* OF4, and the results suggested that the SLH domain-containing protein, other than SlpA, plays an important role in alkaline adaptation via peptidoglycan synthesis (Figures 2, 3).

The CS54 strain, a *csaB* gene deletion mutant of *B. pseudofirmus* OF4, grew as cell clusters in liquid medium and showed a chained morphology (Supplementary Figures S1, S2). Cell clustering also appeared to be more accelerated when cultured at a low shaking speed. These results strongly suggested that cells of the CS54 strain did not separate. A similar extraordinarily chained morphology has also been reported in *csaB* mutant strains of *B. anthracis* (Mesnage et al., 2000). It was reported that the SLH domain-containing PG hydrolase BslO mediates cell separation and is a determinant of *B. anthracis* chain length (Anderson et al., 2011). The putative SLH domain-containing PG hydrolase-encoding genes have been found in the genome of *B. pseudofirmus* OF4 (Table 1), suggesting that the cell separating PG hydrolase is delocalized from the cell wall in the CS54 strain, which causes a defect in cell separation leading to the chained morphology.

It has been reported that the *slpA* mutant RG21 strain has poorer growth than the parental 811M strain under conditions of an extremely high alkaline pH and low Na^+^ concentration, such as pH 11 and 5 mM Na^+^(Gilmour et al., 2000). Therefore, the growth of the CS54 strain was examined at several pH values. To investigate the growth of clustered cells, the amount of DNA was quantified (Figure 2). It has already been confirmed that chromosomal DNA is segregated, even in CS54 cells (Supplementary Figure S3), and the amount of DNA is proportional to the number of cells. There was no significant difference in the amount of DNA among the 811M, RG21, and CS54-R strains at each pH value, but the amount of the CS54 strain at pH 10 or higher was obviously smaller. These results suggest poorer growth of the CS54 strain than the 811M strain in an alkaline environment even under 230 mM Na^±^ concentrations. The CS54 strain showed greater alkaline sensitivity than the RG21 strain, which strongly suggests that SLH domain-containing proteins, other than SlpA, are involved in the alkaline adaptation of *B. pseudofirmus* OF4.

Bands were detected at about 94 kDa in the cell wall protein fractions of the 811M and CS54-R strains by SDS-PAGE, corresponding to the size of the SlpA protein (Figure 5). These bands were also detected in the extracellular protein fractions. It has also been reported that the SLH domain-containing proteins were detected not only in the cell wall of *B. anthracis* but also in the medium (Lunderberg et al., 2013). The bands of about 94 kDa were not detected in the RG21 strain in both fractions, indicating defects of the SlpA protein. In the CS54 strain, no bands of about 94 kDa were detected in the cell wall fraction, and a weak band was detected at about 94 kDa in the extracellular protein fraction, indicating the possibility that SlpA protein is secreted into the medium but is not retained on the cell wall due to CsaB deficiency. In all cases, no bands of about 40 kDa, corresponding to the size of CsaB protein, were detected. Even in *B. anthracis*, CsaB protein was not detected by SDS-PAGE (Lunderberg et al., 2013), suggesting that the CsaB protein expressed at very low levels.

Next, ultrathin section TEM of the cell wall structure of *B. pseudofirmus* OF4 was performed (Figure 3) and the thicknesses of the S-layer, PG, and cell wall (S-layer + PG) were measured from the photograph (Table 4). The 811M and CS54-R strains had the S-layer as the outermost cell envelope component and homogeneous PG layers. The RG21 strain lacked an S-layer, but its PG was not affected, in accordance with the results of a previous study (Gilmour et al., 2000). On the other hand, the CS54 strain lacked an S-layer and its PG was heterogeneously disturbed. These results suggest that SlpA is a major protein of the S-layer and CsaB is necessary not only for *B. anthracis*, but also for *B. pseudofirmus* OF4 in order to maintain the SLH domain-containing proteins on the cell wall.

To investigate PG synthesis of the CS54 strain, the staining patterns of the cells were compared using fluorescent derivatives of the PG-binding antibiotic vancomycin (Figure 4). Bright signals were observed at mid-cell and the poles of the cells of every strain. Similar results have already been reported in *B. halodurans* C-125 and *B. subtilis* (Tiyanont et al., 2006; Fujinami et al., 2011). However, an additional bright signal was observed in the clustered CS54 cells, suggesting that the newly inserted PG was disturbed, which was considered to be responsible for the formation of the heterogeneous PG layer observed in Figure 3. It was suggested that the SLH domain-containing PG hydrolase, which is involved in PG metabolism, is also delocalized from the cell wall of the CS54 strain. It was reported that the cross-linking rate of PG of *B*. *halodurans* C-125 cells grown at pH 7.0 was lower than those grown at pH 10 (Aono and Sanada, 1994), indicating that incomplete PG is responsible for alkaline sensitivity.

The abundance of acidic polymers on the cell wall, such as SlpA of *B. pseudofirmus* OF4, has been thought to prevent hydroxide ion penetration, due to the high anionic charge, and promote proton accumulation around the cell (Krulwich, 1995; Gilmour et al., 2000; Fujinami and Fujisawa, 2010). In order to verify this hypothesis, measurement of the surface potential is necessary. However, it was reported that zeta potential does not accurately represent the surface potential of bacterial cells because of the Smoluchowski equation cannot be applied to a polymer-covered soft particle, such as bacterial cells (Morisaki et al., 1999). Therefore, we attempted to detect EPM (electrophoretic mobility) differences in the cells of each strain. Furthermore, since it is reported that flagella are a key factor that determines cell surface properties in *B. subtilis* (Okuda et al., 2003), we carried out a process to remove flagella from the cells by treatment with a syringe for 30 s before the measurement. As a result, no significant difference in EPM could be detected between the 811M strain and its derivatives, at least under this condition (Supplementary Figure S4).

It is not yet clear how SlpA contributes to alkaline adaptation. However, it was suggested that PG contributed more to alkaline adaptation than SlpA. In *B. subtilis*, PG hydrolases have been identified in cell separation and/or PG synthesis (Fukushima et al., 2006). In future studies, it is necessary to identify SLH domain-containing PG hydrolases involved in cell separation and /or PG synthesis in *B. pseudofirmus* OF4 and to find a PG hydrolase that plays a central role in cell morphology and/or alkaline adaptation.

## Author contributions

SF and MI designed research. SF performed research. SF and MI analyzed data and wrote the paper.

### Conflict of interest statement

The authors declare that the research was conducted in the absence of any commercial or financial relationships that could be construed as a potential conflict of interest.
