# Supplementary material for: The Surface Layer Homology Domain-Containing Proteins of Alkaliphilic Bacillus pseudofirmus OF4 Play an Important Role in Alkaline Adaptation via Peptidoglycan Synthesis
Source: Front Microbiol. 2018 May 1;9:810. doi: 10.3389/fmicb.2018.00810 (PMC5938343; doi:10.3389/fmicb.2018.00810)
Supplement: Supplementary file 1 [file Image_1.PDF]

Supplementary Material

The surface layer homology domain-containing proteins of alkaliphilic *Bacillus pseudofirmus* OF4 play an important role in alkaline adaptation via peptidoglycan synthesis

Shun Fujinami[1,2,*] and Masahiro Ito [1,3]

1 Bio-Nano Electronics Research Centre, Toyo University, Kawagoe, Saitama, Japan

2 Department of Chemistry, College of Humanities and Sciences, Nihon University, Setagaya-ku, Tokyo, Japan

3 Graduate School of Life Sciences, Toyo University, Oura-gun, Gunma, Japan

*Correspondence: Shun Fujinami, Ph.D.

Mailing address: Department of Chemistry, College of Humanities and Sciences, Nihon University, Setagaya-ku, Tokyo, Japan.

Tel & Fax: +81-3-5317-9735

E-mail: fujinami.shun@nihon-u.ac.jp

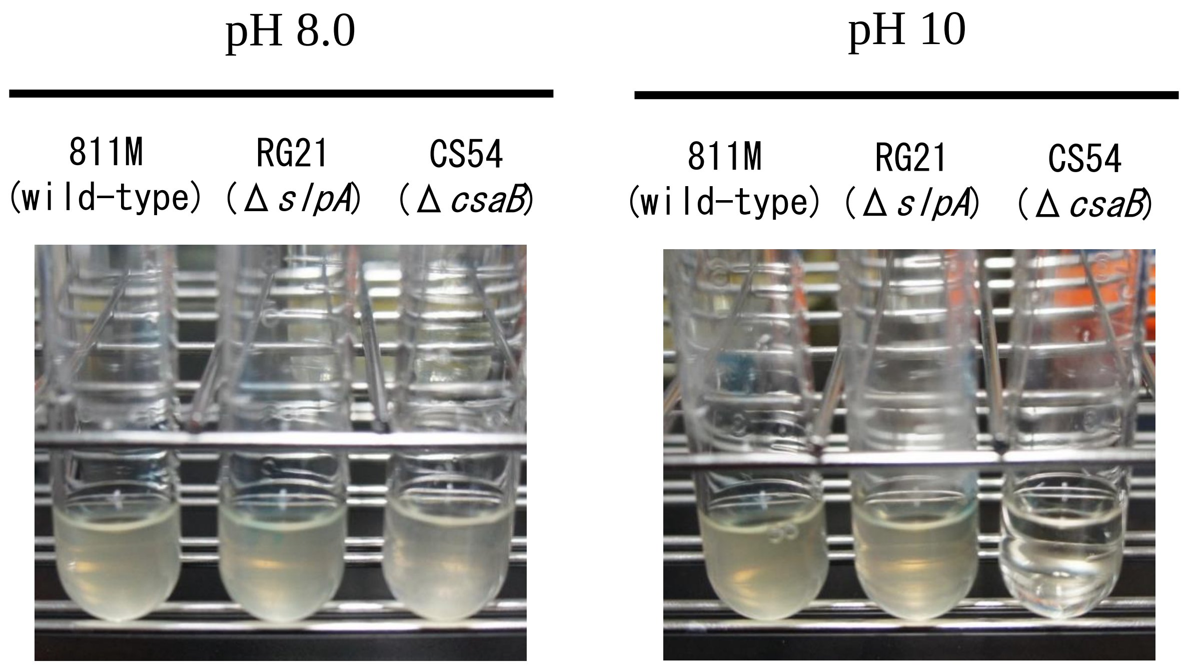

Supplementary Figure S1. Observation of the liquid-grown *B. pseudofirmus* OF4-811M and its derivatives.

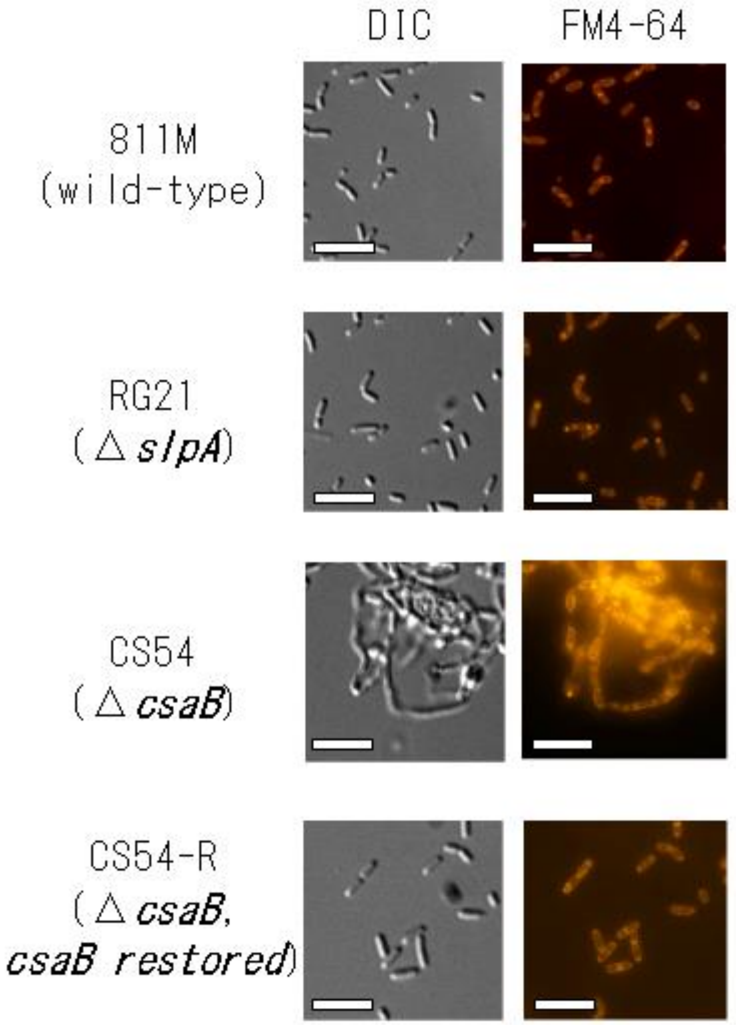

Supplementary Figure S2. Microscopic observation of the cytoplasmic membrane of *B. pseudofirmus* OF4-811M and its derivatives.

Abbreviations: DIC, differential-interference contrast microscopy; FM4-64, fluorescence microscopy for cytoplasmic membrane stained with FM4-64. All photographs are of the same magnification. Scale bar: 5 μm.

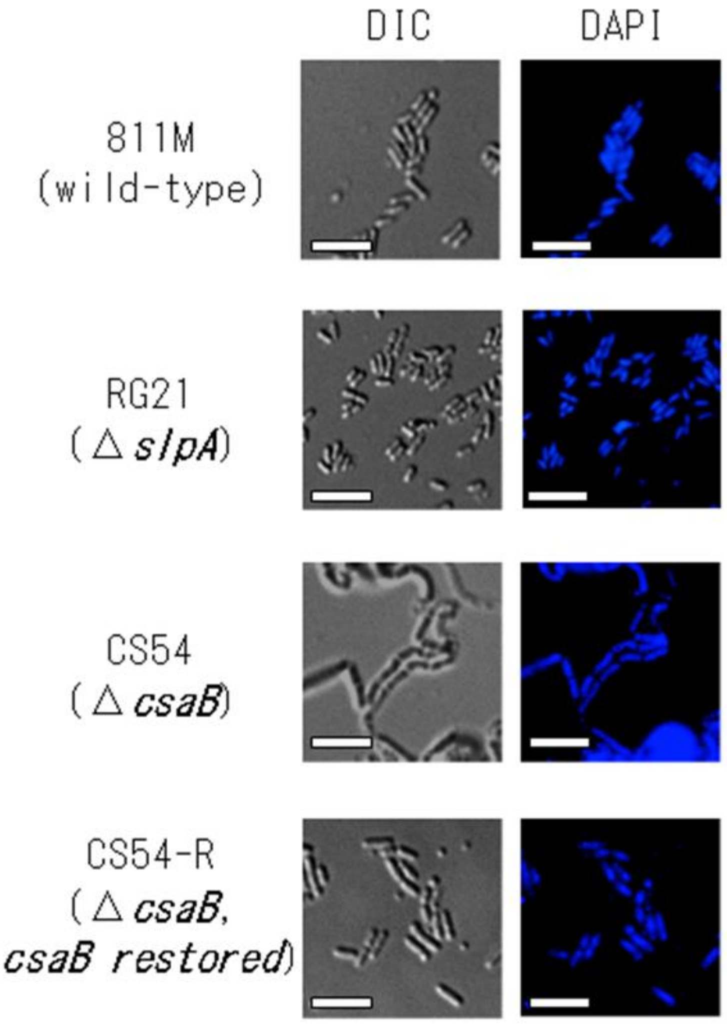

Supplementary Figure S3. .Microscopic observation of the nucleoids of *B. pseudofirmus* OF4-811M and its derivatives. Abbreviations: DIC, differential-interference contrast microscopy; DAPI, fluorescence microscopy for nucleoid stained with DAPI. All photographs are of the same magnification. Scale bar: 5 µm.

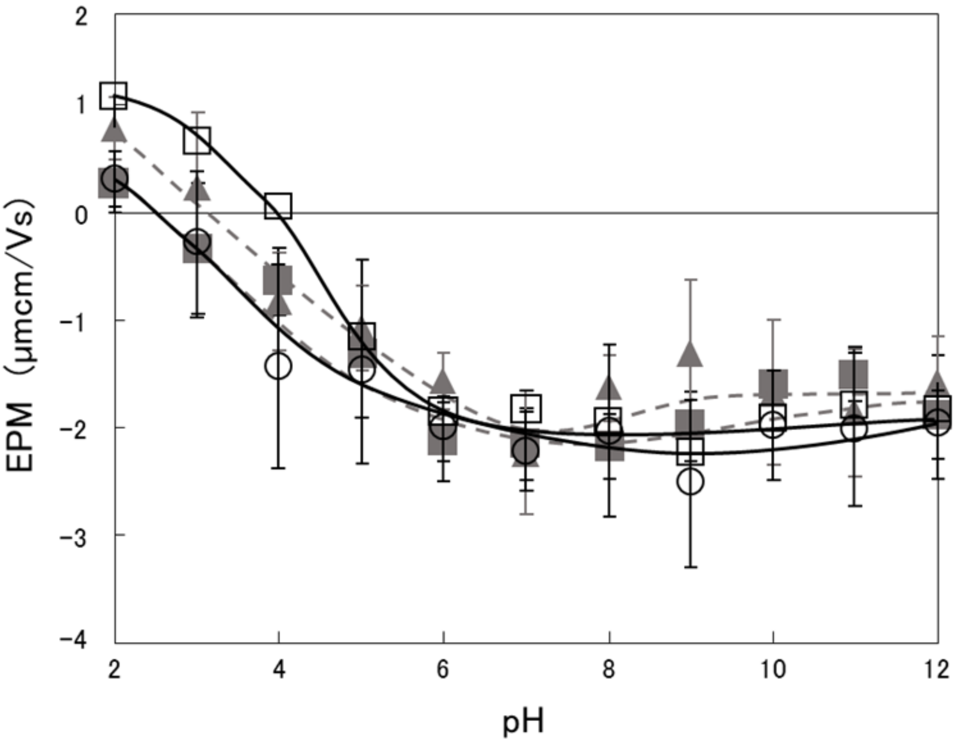

Supplementary Figure S4. Electrophoretic mobility (EPM) of *B. pseudofirmus* OF4-811M and its derivative cell. The black solid line and open circles show data for the 811M strain, the gray dashed line and filled triangles show data for the RG21 strain, the black solid line and open squares show data for the CS54 strain, and the gray dashed line and filled squares show data for the CS54-R strain.
